# Outcomes of Using Nasolabial Flap for Orofacial Reconstruction: A Retrospective Descriptive Case Series

**DOI:** 10.7759/cureus.102242

**Published:** 2026-01-25

**Authors:** Kaoru Murakami, Chikashi Minemura, Hiroaki Morihara, Takafumi Nobuchi, Yuriko Toeda, Yosuke Hirata, Hidetaka Yokoe

**Affiliations:** 1 Department of Oral and Maxillofacial Surgery, National Defense Medical College, Tokorozawa, JPN

**Keywords:** local flaps, nasolabial flap, orofacial reconstruction, pedicled flap, random-pattern flap, tunneled island flap

## Abstract

Purpose

This retrospective descriptive study aimed to evaluate the clinical outcomes, complication rates, and flap survival of the nasolabial flap used for reconstruction of various orofacial soft-tissue defects, with particular attention to the flap design (tunneled island flap vs pedicled flap) and the impact of facial artery ligation.

Patients and methods

A total of 20 cases at the Department of Oral and Maxillofacial Surgery, National Defense Medical College Hospital, between 2017 and 2025, in which the nasolabial flap was used to reconstruct soft-tissue defects, resulting from oral cancer, jaw osteonecrosis, soft-tissue defects due to trauma, and oronasal fistula, were retrospectively reviewed after obtaining ethical approval from the ethics committee.

Results

A total of 17 cases involved intraoral reconstruction using a tunneled island flap. Meanwhile, the pedicled flap was used in three cases for lip reconstruction. In all cases, the flap survived, and satisfactory functional and esthetic outcomes were achieved. In three cases, flap resection was required after reconstruction, and only one (5%) patient developed partial flap dehiscence. Two (10%) patients exhibited distal flap necrosis, both of which involved random-pattern and tunneled island flaps in patients who had their facial artery ligated, but the flap’s viability was ultimately good. All cases of orofacial reconstruction using the nasolabial flap during the study period were included.

Conclusions

Based on our study, the nasolabial flap is a reliable reconstructive method for orofacial defects. However, we believe that cautious attention to blood supply is needed in cases involving tunneled island flaps where the facial artery is ligated.

## Introduction

Local flaps are used for reconstructing soft-tissue defects in the orofacial region, addressing misalignment of anatomical landmarks caused by suturing that can result in aesthetic or functional issues. In recent years, microsurgical free flaps have been frequently used for the reconstruction of orofacial soft tissues. Contemporary reconstruction increasingly integrates virtual surgical planning to optimize flap design and outcomes [1]. However, even in cases in which reconstruction with a free flap is feasible, local flaps should be utilized if sufficient reconstruction can be achieved with them or if local flaps can yield superior color and texture matching. In particular, this is important when reconstructing facial skin or lip defects attributed to trauma or tumor resection, where both aesthetics and function must be cautiously considered. Compared with free flaps, local facial flaps provide better color and texture matching, and are less invasive for patients undergoing facial reconstruction. The nasolabial flap is a local facial flap created near the nasolabial fold. The nasolabial flap is technically straightforward, has a stable blood flow, and is associated with few complications. This flap can be used to reconstruct different areas such as the nose, lips, cheeks, and oral cavity [2]. The earliest description of the nasolabial flap can be traced back to Sushruta around the 6th century BC [3]. Thereafter, it has been utilized in modern medicine for orofacial soft-tissue reconstruction. In our institution, the nasolabial flap was actively used to reconstruct orofacial soft-tissue defects caused by malignant tumors, osteonecrosis, trauma, and oronasal fistula, achieving favorable outcomes. Only a few reports have examined the application of the nasolabial flap for various orofacial reconstructions. The current study aimed to evaluate the clinical outcomes, complication rates, and flap survival of the nasolabial flap used for reconstruction of various orofacial soft-tissue defects, with particular attention to flap design, tunneled island flap vs pedicled flap, and the impact of facial artery ligation.

## Materials and methods

Study details

A retrospective study was conducted on 20 cases at the Department of Oral and Maxillofacial Surgery, National Defense Medical College Hospital, between 2017 and 2025, in which the nasolabial flap was used to reconstruct soft-tissue defects resulting from oral cancer, jaw osteonecrosis, soft-tissue defects due to trauma, and oronasal fistula. Data including age, sex, diagnosis, defect site, with/without smoking, with/without anticoagulation therapy, antimicrobial prophylaxis, type of flap used (unilateral or bilateral flap, random- or axial-pattern flap, pedicled or island flap, or inferiorly or superiorly based flap), type of procedure applied (with/without facial artery ligation, with/without pedicle resection), flap survival, and presence of dehiscence, surgical site infection, mouth opening limitations, and follow-up period were reviewed from the medical records of the patients. The Ethics Committee of the National Defense Medical College approved this study (approval no. 5246).

Surgical technique

All surgeries were performed by one senior oral and maxillofacial surgeon with more than 15 years of clinical experience. The nasolabial flap was created near the nasolabial fold. Two methods were used to elevate the flap. The first technique involved the facial artery-which runs subcutaneously along the nasolabial fold-within the flap, elevating it as an axial-pattern flap. The second technique excluded the facial artery within the flap and instead elevated it as a random-pattern flap via the subcutaneous fat layer. Generally, the random-pattern method is more commonly used. Due to the rich subdermal vascular network surrounding the nasolabial fold, the flap is safe to elevate to a length-to-width ratio of up to 4:1 [4]. Only one case required a particularly thick and long flap for lip reconstruction performed using the axial-pattern method. Meanwhile, the random-pattern method was utilized in the remaining 19 cases. The medial edge of the flap was aligned with the nasolabial fold, and the flap was designed to have a width of 15-25 mm. A pedicled flap was used to reconstruct extraoral defects. For intraoral reconstruction, a transbuccal tunnel was created to transfer the tunneled island flap intraorally, ensuring not to damage the facial artery. Hence, special precautions were taken to create a sufficiently wide transbuccal tunnel to prevent flap compression. In the axial-pattern flap case, the course of the facial artery was identified preoperatively on doppler ultrasonography. The flap was designed to include the facial artery, along with the surrounding mimetic muscles, which were elevated together with the flap. In the random-pattern flap cases, the flap was elevated via the subcutaneous fat layer while ensuring sufficient thickness for reconstruction. The donor site of the flap was closed primarily, and the triangular area at the base of the flap was de-epithelialized at that time. In the three cases of intraoral reconstruction, the flap passing through the transbuccal tunnel was resected three weeks postoperatively to achieve optimal cosmetic and functional outcomes at the reconstructed site.

## Results

In total, 20 cases in which the nasolabial flap was used for orofacial soft-tissue reconstruction were retrospectively reviewed (Table 1).

**Table 1:** Summary of outcomes SCC: squamous cell carcinoma, ACC: adenoid cystic carcinoma, MRONJ: medication-related osteonecrosis of the jaws, MEC: mucoepidermoid carcinoma, PFD: posttraumatic facial defect, ORN: osteoradio necrosis.

Patient no.	Age (years)	Sex	Diagnosis	Defect site	Smoking	Anticoagulation therapy	Antimicrobial prophylaxis	Unilateral or bilateral flap	Random- or axial-pattern flap	Pedicled or island flap	Inferiorly or superiorly based flap	Facial artery ligation	Pedicle resection	Flap survival	Dehiscence	Surgical site infection	Limitations in mouth opening	Follow-up (month)
1	70	M	Oronasal fistula	Palate	No	No	Cefmetazole 48 hr	Unilateral	Random	Island	Superiorly	No	Yes	Complete	No	No	No	62
2	76	F	SCC of the lower gingiva	Lower gingiva	No	No	Cefmetazole 48 hr	Unilateral	Random	Island	Inferiorly	No	No	Complete	No	No	No	104
3	74	F	ACC of the buccal mucosa	Buccal mucosa	No	No	Cefmetazole 24 hr	Unilateral	Random	Island	Superiorly	No	No	Complete	No	No	No	99
4	78	F	MRONJ of the mandible	Lower gingiva	No	No	Cefmetazole 120 hr	Unilateral	Random	Island	Inferiorly	No	No	Complete	No	No	No	24
5	67	M	SCC of the lower gingiva	Lower gingiva	No	No	Sulbactam /Ampicillin 48 hr	Unilateral	Random	Island	Inferiorly	Yes	No	Distal necrosis	No	No	No	60
6	71	M	MEC of the palate	Palate	Yes	No	Cefmetazole 48 hr	Unilateral	Random	Island	Superiorly	No	No	Complete	No	No	No	79
7	77	F	SCC of the lower gingiva	Lower gingiva	No	No	Sulbactam /Ampicillin 48 hr	Unilateral	Random	Island	Inferiorly	Yes	No	Complete	No	Yes	No	70
8	88	F	SCC of the lower gingiva	Lower gingiva	No	Yes	Cefmetazole 48 hr	Unilateral	Random	Island	Inferiorly	No	No	Complete	No	No	No	36
9	79	M	SCC of the lower lip	Lower lip	No	No	Cefmetazole 48 hr	Unilateral	Axial	Pedicled	Inferiorly	No	No	Complete	No	No	No	54
10	74	M	SCC of the floor of the mouth	Floor of the mouth	Yes	No	Cefmetazole 72 hr	Bilateral	Random	Island	Inferiorly	Yes	Yes	Complete	No	No	No	49
11	82	F	SCC of the lower gingiva	Lower gingiva	No	No	Cefmetazole 48 hr	Unilateral	Random	Island	Inferiorly	Yes	No	Complete	No	No	No	22
12	43	M	SCC of the lower gingiva	Lower gingiva	Yes	No	Cefmetazole 48 hr	Unilateral	Random	Island	Inferiorly	No	No	Complete	No	Yes	No	3
13	28	M	PFD of the lower lip	Lower lip	No	No	Cefmetazole 96 hr	Unilateral	Random	Pedicled	Inferiorly	No	No	Complete	No	No	No	18
14	75	F	SCC of the lower gingiva	Lower gingiva	Yes	No	Cefmetazole 48 hr	Unilateral	Random	Island	Inferiorly	No	No	Complete	No	No	No	28
15	64	M	SCC of the upper gingiva	Upper gingiva	No	No	Cefmetazole 48 hr	Unilateral	Random	Island	Superiorly	No	Yes	Complete	No	No	No	26
16	82	F	SCC of the lower gingiva	Lower gingiva	No	No	Cefmetazole 48 hr	Unilateral	Random	Island	Inferiorly	No	No	Complete	No	No	No	16
17	80	F	SCC of the buccal mucosa	Buccal mucosa & lower lip	No	No	Cefmetazole 48 hr	Unilateral	Random	Pedicled	Inferiorly	Yes	No	Complete	No	No	No	5
18	80	F	SCC of the lower gingiva	Lower gingiva	No	No	Cefmetazole 48 hr	Unilateral	Random	Island	Inferiorly	Yes	No	Distal necrosis	No	Yes	No	12
19	74	F	ORN of the mandible	Lower gingiva	No	No	Cefmetazole 96 hr	Unilateral	Random	Island	Inferiorly	No	No	Complete	No	No	No	8
20	79	F	SCC of the lower gingiva	Lower gingiva	No	No	Cefmetazole 48 hr	Unilateral	Random	Island	Inferiorly	No	No	Complete	Partial	No	No	8

**Figure 1:**
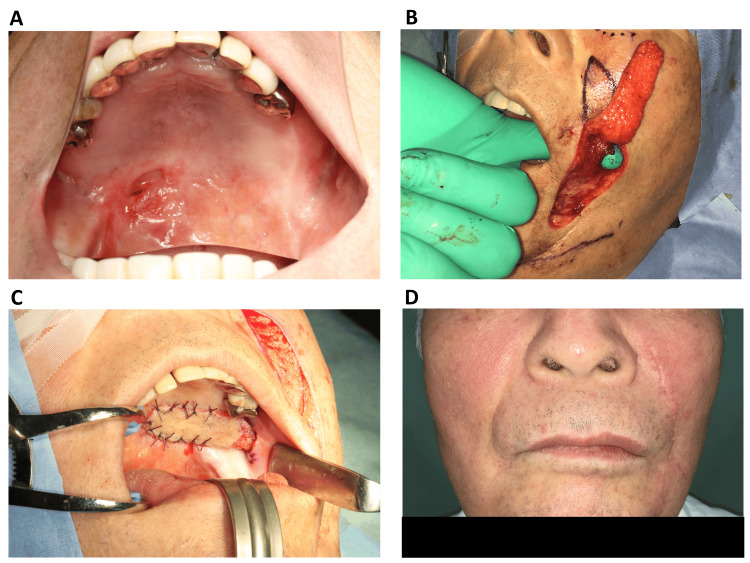
Pre- and postoperative images A) Preoperative view of the oronasal fistula (mirror image); B) An elevated nasolabial flap on the left side and the transbuccal tunnel was created to transfer the tunneled island nasolabial flap intraorally; C) Covering the oronasal fistula by the tunneled island nasolabial flap; D) Two-month postoperative frontal view.

Of these, 17 involved intraoral reconstruction using the tunneled island flap. Meanwhile, the pedicled flap was used in three cases involving lip reconstruction. The breakdown of the underlying diseases was as follows: oral cancer resection (n=16); mandibular osteonecrosis (n=2); traffic accident-related injury (n=1); and oronasal fistula (n=1) (Figure 1).

**Figure 2:**
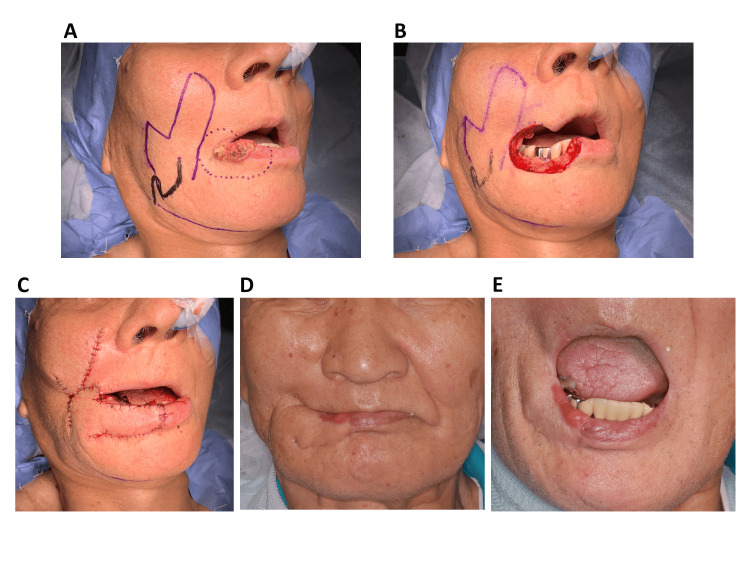
Pre- and post-surgical images A) Presurgical marking modified the pedicled nasolabial flap as an axial pattern, and the dotted line shows the surgical margin surrounding lip squamous cell carcinoma; B) Lip squamous cell carcinoma resection; C) Reconstruction of lip defect by the pedicled nasolabial flap; D) Four-year postoperative frontal view showed good color and texture match; E) Four-year postoperative view revealed no limitations in mouth opening.

The reconstruction sites were the mandibular gingiva in 12 cases, the lip in three, the palate in two, the buccal mucosa in two, and the floor of the mouth in one. The flap was unilateral in 19 cases and bilateral in one. The random-pattern flap was used in 19 cases and the axial-pattern flap in one. One axial-pattern flap case involved lip reconstruction in a patient with lip cancer. In this case, a modified flap design was used to reconstruct both the upper and lower lips, including the oral commissure. Thus, the random-pattern flap was considered unreliable, and the axial-pattern flap was selected (Figure 2).

Three cases required flap resection after reconstruction. Partial flap dehiscence was noted only in one case. However, it did not cause any clinical problems. This partial flap dehiscence occurred on postoperative day 17, but healed completely by postoperative day 34 without any additional intervention. Surgical site infection (SSI) was observed in the neck of three patients who underwent neck dissection for mandibular gingival cancer. Nevertheless, there were no cases of SSI at the donor site, and all flaps had good viability. In six cases, the facial artery was ligated during neck dissection. Among them, two developed distal necrosis. These two distal necrosis cases occurred on postoperative day 10 and healed within one month after irrigation alone. The flap’s viability was ultimately good. There were no cases of postoperative trismus. All cases of orofacial reconstruction using nasolabial flap during the study period were included. All cases received nasogastric feeding for at least seven days postoperatively.

## Discussion

The key characteristics of the nasolabial flap include the ability to choose either a random-pattern or axial-pattern design based on the reconstruction site and area, adjust the flap size, select between inferiorly and superiorly based flaps, and decide between unilateral and bilateral designs [2,5,6]. Generally, a random-pattern flap, utilizing the rich subdermal vascular plexus of the facial skin, is selected as the nutrient vessel. The axial pattern, which uses the facial artery as the nutrient vessel, is indicated if more reliable blood flow is required due to the flap thickness [2]. Even in cases where the facial artery had been ligated during neck dissection, several reports have shown that the nasolabial flap is still viable and highly reliable due to a rich collateral vascular plexus [7-9]. However, Rahpeyma et al. have revealed that if the facial artery is ligated, inferiorly based nasolabial flaps with an axial pattern are not indicated. However, infraorbital-based nasolabial flaps fed by the infraorbital artery can be used [2]. There have been no cases involving inferiorly based nasolabial flaps with an axial pattern in patients whose facial artery had been ligated. Nevertheless, we considered the assertion by Rahpeyma et al. to be reasonable. In our study, distal necrosis of the nasolabial flap was observed in two (10%) cases, both of which involved random-pattern flaps in patients who had their facial artery ligated. We believe that the reliability of random-pattern nasolabial flaps remains high even if the facial artery is ligated. Nevertheless, greater attention must be given to the blood supply compared with cases in which the facial artery is intact. Neck dissection was performed for mandibular gingival cancer in three cases. However, none of these cases had SSI at the flap donor site, thereby indicating that this flap is resistant to infection. This resistance is likely caused by a rich blood supply, a characteristic of the nasolabial flap. Compared with pedicled flaps, we believe that tunneled island flaps require even greater attention in terms of maintenance of blood supply. To prevent compression of the flap as it passes through the transbuccal tunnel, a sufficiently wide transbuccal tunnel is created. At this time, caution must also be taken not to injure the facial artery adjacent to the transbuccal tunnel. In addition, when designing a pedicled flap, we consider the pivot point placement to decrease the risk of flap twisting. Lazaridou et al. have found that tunneled island flaps have a higher rate of flap-related complications than pedicled flaps. In particular, de-epithelialization of the flap’s pedicle increases versatility and the ability to reach further. However, caution is required to prevent ischemic complications, as the pedicle may become excessively stretched [7]. The regions that can be reconstructed with a nasolabial flap are extensive, and they include the lower eyelid, nose, white lip, cheek, buccal mucosa, hard palate, gingiva, and floor of the mouth [2]. For facial reconstruction sites adjacent to the flap harvesting area, a pedicled flap is used. If the reconstruction site is not adjacent, the intervening skin can be excised, or a subcutaneous pedicle flap can be applied. For oral cavity reconstruction, the tunneled island flap is created by passing through the transbuccal tunnel. If necessary, the flap is resected after successful engraftment. In our cases, 17 oral cavity reconstructions were performed using tunneled island flaps, and three lip reconstructions were conducted using pedicled flaps. In three cases where the flap severely impaired oral vestibule or caused trismus, the flap was resected after the reconstruction. In cases where the buccal mucosa is not included in the reconstruction area and the reconstruction site is not contiguous with the transbuccal tunnel, our policy is that flap resection will be indicated, and resection is performed three weeks after reconstruction. In our study, the flap-related complications were limited to distal necrosis (10%) and partial dehiscence (5%). Chitlangia et al. reported 12.5% of partial necrosis cases in patients (n=40) who underwent surgery using the nasolabial flap [10]. The nasolabial flap is a reliable local flap that is associated with few complications. However, some studies have reported about buccal cysts caused by insufficient de-epithelialization in tunneled island flaps [7,11]. Therefore, caution is required. In men, the use of the tunneled island flap for intraoral reconstruction may cause issues in facial hair within the oral cavity. In the fifth patient in our study, facial hair was noted after intraoral reconstruction, and the problem was resolved by de-epithelialization after the flap had been taken. Bhambri et al. have revealed that the nasolabial flap is an excellent surgical option for the closure of recurrent oroantral fistula, particularly in patients with chronic osteomyelitis [12]. In our study, good outcomes were also achieved using the nasolabial flap for the closure of oronasal fistula and decortication for osteonecrosis. Lemound et al. have reported that the nasolabial flap can be a highly reliable option for bone wound coverage, with less morbidity than microsurgical free flaps and better long-term results compared with mucoperiosteal flaps [13]. The nasolabial flap is an old local flap used for the reconstruction of orofacial soft tissues. Over time, various modifications have been introduced to expand its indications [3,4]. The nasolabial flap was originally created on the same side as the reconstruction site. However, some reports have shown that newly developed contralateral nasolabial flaps have favorable outcomes, thereby expanding the indications for this flap [14]. The limitations of this study include retrospective design, small sample size, single-center study, and the absence of other local flaps or micro-surgical free flaps as control groups.

## Conclusions

The nasolabial flap is a reliable reconstructive method for soft-tissue defects in the orofacial region that are associated with oral cancer, jaw osteonecrosis, orofacial trauma, and oronasal fistula. In particular, when reconstructing the lip, the nasolabial flap’s excellent color and texture matching provides significant advantages in terms of cosmetic outcomes. Considering its simplicity, low invasiveness, and reliable blood supply, the nasolabial flap should be considered as an option for soft-tissue reconstruction in the orofacial region. However, we believe that cautious attention to blood supply must not be disregarded in cases involving tunneled island flaps where the facial artery is ligated.
